# Supplementary material for: A network modeling approach to elucidate drug resistance mechanisms and predict combinatorial drug treatments in breast cancer
Source: Cancer Converg. 2017 Dec 29;1(1):5. doi: 10.1186/s41236-017-0007-6 (PMC5876695; doi:10.1186/s41236-017-0007-6)
Supplement: Supplementary file 1 — Details of the model: full node names, regulatory functions and update information. (DOCX 38 kb) [file 41236_2017_7_MOESM1_ESM.docx]

Explanation of the node names

ESR1 – the transcript of the *ESR1* gene, which encodes the estrogen receptor

ER- estrogen receptor status of the cell

HER2- human epidermal growth factor receptor 2 status,

HER3- human epidermal growth factor receptor 3,

HER2/HER3 – heterodimer of HER2 and HER3

IGF1R – insulin-like growth factor 1 receptor (IGF1R) and insulin receptor (INSR)

IGF1R_T – transcript of IGF1R or INSR

PIP3 – phosphatidylinositol – (3,4,5) –triphosphate

PI3K – phosphatidylinositol – 4,5- biphosphate 3-kinase, encoded by the gene *PIK3CA*

PTEN – phosphatase and tensin homolog

AKT – protein kinase B

RAS – Ras family small GTPase,

MAPK – merged node that includes RAF- rapidly accelerated fibrosarcoma kinase family,

MEK- mitogen-activated protein kinase kinase, and ERK- extracellular signal-regulated kinase

FOXA1 – forkhead box protein A1

PBX1 – pre-B cell leukemia transcription factor 1

KMT2D – histone-lysine N methyltransferase 2D

MYC - transcription factor encoded by the proto-oncogene *c-myc*

PDK1 – 3-phosphoinositide dependent protein kinase 1, encoded by the gene *PDPK1*

PDK1_pm - 3-phosphoinositide dependent protein kinase 1 localized in the plasma membrane

SGK1 – serum and glucocorticoid-induced kinase 1

SGK1_T – transcript of serum and glucocorticoid-induced kinase 1

PIM – Pim-1 Proto-Oncogene, Serine/Threonine Kinase (PIM1), Pim-2 Proto-Oncogene, Serine/Threonine Kinase (PIM2), and Pim-3 Proto-Oncogene, Serine/Threonine Kinase (PIM3).

cycD/CDK4/6 – complex formed by cyclin D1 and cyclin dependent kinase 4/6,

cycE/CDK2 – complex formed by cyclin E and cyclin dependent kinase 2,

Rb – retinoblastoma family proteins

E2F- transcriptional activator members of the E2F family

p21 – WAF1/CIP1, cyclin-dependent kinase inhibitor 1, encoded by the gene *CDKN1A*

p27- KIP1, cyclin-dependent kinase inhibitor 1B, encoded by the gene *CDKN1B*

mTORC1 - mechanistic target of Rapamycin complex 1

mTORC2_pm - mechanistic target of Rapamycin complex 2 localized in the plasma membrane

mTORC2 - mechanistic target of Rapamycin complex 2 not localized in the plasma membrane and possibly localized in the mitochondria.

TSC – tuberous sclerosis complex 1 (TSC1) and tuberous sclerosis complex 2 (TSC2)

PRAS40 - proline-rich Akt substrate of 40 kDa, a component of mTORC1

FOXO3- forkhead box O3 protein

FOXO3_Ub – forkhead box O3 protein degraded through the ubiquitin proteasome pathway

S6K – p70 ribosomal S6 kinase

EIF4F - eukaryotic initiation factor 4A(EIF4A), eukaryotic initiation factor 4G (EIF4G), and Eukaryotic Translation Initiation Factor 4E Binding Protein 1 (4EBP1)

Translation – processes related to ribosome translation, cap-dependent translation

Proliferation – focusing on two requirements: cell cycle transition from G1 to S, and protein translation

BCL2- B-cell lymphoma 2 protein, anti-apoptotic family representative

BCL2_T – B cell lymphoma 2 transcript

MCL1 – BCL2 family apoptotic regulator, anti-apoptotic

BAD- Bcl-2-associated death promoter, apoptosis sensitizer family representative

BIM- Bcl-2-like protein 11, apoptosis activator family representative

BIM_T – transcript of BCl-2-like protein 11

Alpelisib – Isoform-specific drug inhibitor of PI3K alpha, also known as BYL719

Fulvestrant – drug inhibitor of the estrogen receptor, a type of selective estrogen receptor degrader (SERD)

Neratinib – drug inhibitor of EGFR and HER2

Palbociclib – drug inhibitor of CDK4/6

Everolimus) – mTORC1 and mTORC2 inhibitor

Trametinib – drug inhibitor of MAPK signaling (specifically, of MEK1 and MEK2)

Ipatasertib – drug inhibitor of AKT

Explanation of the node states

To maximize the parsimony of the network, we aim to represent each gene product with the minimal number of nodes. This can be a single node if there is a single main mode of regulation of that gene (e.g. transcriptional). If there is both transcriptional and (post)translational regulation, we have a node for the transcript (marked by the subscript “T”) and a separate node for the protein. In the case of PDK1 and mTORC2 there is evidence of two different pools (localizations) that are regulated differently (i.e. the plasma membrane localized PDK1 and mTORC2 are regulated by PIP3) and have different effects (i.e. one localization is mainly responsible for the regulation of AKT, while the other is mainly responsible for the regulation of SGK1). For this reason we define two nodes for PDK1 and mTORC2. Finally, the post-translational modifications of FOXO3 can be categorized into faster processes (phosphorylation-induced translocation) and the slower process of phosphorylation-induced proteolytic degradation. Because of this reason we define a separate node, FOXO3_Ub, and update it with a lower probability.

For the 34 binary nodes, the node name implicitly represents the active state of the node. For example, RAS refers to the GTB-bound active form of the RAS GTPase. If a protein is activated by phosphorylation (such as AKT, S6K, SGK1), the node name refers to the phosphorylated form, and the regulatory function expresses the condition under which the protein is activated (e.g. AKT is phosphorylated and activated by PDK1 and mTORC2). If a protein is active if unphosphorylated (such as p21, p27, FOXO3, BAD, BAX), the node name refers to the unphosphorylated form; the network and the regulatory function will reflect phosphorylation as a negative regulation of this node.

The node states of the receptors ER and HER2 reflect the receptor expression status of the cell; i.e. an ER+ cell would have ER=1.

Multi-level nodes characterize the receptor tyrosine kinases IGFR1, HER3, the HER2/HER3 heterodimers, the PI3K pathway, RAS and the MAPK pathway, the ER pathway, the majority of the cell cycle related proteins (e.g. cyclin D, Rb, E2F), and the outcome nodes proliferation and apoptosis. For these 16 multi-level nodes, separate regulatory functions are given for the basal activity, activity level 2 (denoted by “_2”), and higher activity levels if they exist. The regulatory functions of the higher activity levels reflect a more stringent condition; thus when a node is activated at a level i>1, it is also activated at level i-1.

In the case of Rb, which is active in the unphosphorylated form, we introduce a multi-level node pRb to denote the (hyper)phosphorylated form of Rb.

The output nodes “Proliferation” and “Apoptosis” reflect the propensity of a cell to commit to cell cycle progression, and programmed cell death, respectively. The higher the node level, the stronger this propensity is. In order to facilitate comparison, we introduce normalized variables “Proliferation_norm_” and “Apoptosis_norm_”. The normalization is exponentially weighted following the formula$x_{norm}\left( x=i \right)=\frac{1}{2^{n-i}}$ , where i=1, ..n, n is the maximal activity level of node *x*, and $x_{norm}\left( x=0 \right)=0$. When performing multiple simulations we report the average of the normalized proliferation and apoptosis values over the ensemble of simulations. These values can be loosely interpreted as estimates of the fraction of a simulated cell population that is undergoing cell cycle progression, or is on the path toward apoptosis, respectively. Rather than interpreting the values themselves, we focus on the sign of the change (i.e. increase or decrease) in these values following an intervention or perturbation.

Regulatory functions in the model

The 10 nodes that are not regulated by other network nodes or by drugs (BCL2_T, BIM_T, ER, IGF1R_T, HER3_T, PBX1, PDK1, PIM, PTEN, SGK1_T) are assumed to maintain their initial state. The regulatory function of these nodes is of the type $f_{X}= \sigma_{X}$, meaning that the future state of node X equals its current state. We do not explicitly indicate these regulatory functions. We do indicate the regulatory functions of the three source nodes that are regulated by drugs (CDK4/6, HER2, mTORC2).

In the regulatory functions that follow for simplicity we represent the node state by the node name (i.e. we use X instead of $\sigma_{X}$).

f_IGF1R_=IGF1R_T or (HER2 and FOXO3)

f_IGF1R_2_=(IGF1R_T or (HER2 and FOXO3)) and not S6K

A basal activity of IGF1R is achievable due to an intrinsic transcript level or by transcriptional upregulation by FOXO3 (PMID: 21215704). The transcriptional upregulation of IGFR1 by FOXO3 depends on the presence of HER2, since it has been found in HER2^+^ cells (PMID: 21215704). In order to activate IGF1R at a higher level S6K must be absent, as S6K indirectly inhibits IGF1R through IRS1 (PMID: 16452206).

f_HER3_T_= HER3_T

f_HER3_=FOXO3 or HER3_T

f_HER3_2_=FOXO3

FOXO3 transcriptionally activates HER3 above its intrinsic level (PMID: 21215704).

f_mTORC2_=mTORC2 and not Everolimus

mTORC2 maintains its activity unless inhibited by Everolimus.

f_SGK1_=SGK1_T and PDK1 and mTORC2

Active SGK1 protein needs to be translated from its transcript and needs to be double phosphorylated by PDK1 and mTORC2 (PMID: 20027184, PMID: 18925875).

f_HER2_= HER2 and not Neratinib

f_HER2/HER3_= HER2 and (HER3 or HER3_2) and not Neratinib

f_HER2/HER3__2= HER2 and ((HER3 and not MAPK_2) or HER3_2) and not Neratinib

HER2 forms heterodimers with HER3 (PMID: 11252954), unless inhibited by Neratinib. Strong activation of MEK inhibits the activity of the heterodimer (PMID: 22552284). We assume that high activation of HER3 is able to overcome the inhibition by MAPK. This is supported by the observation that in HER2+ breast cancer AKT inhibition leads to an increase in MAPK activity (PMID:24436048). This increase is mediated by the activation of FOXO3, which transcriptionally upregulates HER3. For the increased MAPK activity to be compatible with HER3 and HER2/3 heterodimer activity, a sufficiently high level of HER3 must be able to overcome MAPK inhibition.

f_RAS_=IGF1R or IGF1R_2 or HER2/HER3 or HER2/HER3_2

f_RAS_2_=HER2/HER3 or HER2/HER3_2

f_RAS_3_=HER2/HER3_2

IGF1R and HER2/HER3 activate RAS (PMID: 17496910, PMID: 21531565).

f_MAPK_=(RAS or RAS_2 or RAS_3) and (PIP3 or PIP3_2) and (not Trametinib or RAS_3)

f_MAPK_2_=(RAS_2 or RAS_3) and PIP3 and (not Trametinib or RAS_3)

RAS activates RAF by translocating it to the plasma membrane, where protein kinases phosphorylate and activate RAF (PMID: 17496910). RAF activates MEK by phosphorylation (PMID: 8325833, PMID: 17496910). PIP3 can indirectly activate MEK (PMID 24327733); the importance of this mechanism (and the use of the “and” operator) is supported by the fact that PI3K inhibition leads to rapid dowregulation of ERK phosphorylation (PMID: 24436048). ERK is activated by double phosphorylation by MEK (PMID: 2032290). Higher activity of RAS yields higher activation of the MAPK pathway, and the highest RAS activity can overcome the inhibitory effect of Trametinib. This rule is consistent with the observation that drug inhibition of MEK in HER2+ cells leads to increased HER2/HER3 heterodimer formation and to reactivation of the MAPK pathway 24 hours after drug treatment (PMID: 22552284, PMID: 24436048).

f_PI3K_=(IGF1R or IGF1R_2 or HER2/HER3 or HER2/HER3_2 or RAS or RAS_2 or RAS_3) and (not Alpelisib or HER2/HER3_2)

f_PI3K_2_= HER2/HER3_2 and not Alpelisib

PI3K is activated by IGF1R, the HER2/HER3 heterodimer or RAS (PMID: 16452206, PMID: 11252954, PMID: 12853564, PMID: 8052307). It is inhibited by the drug Alpelisib (also known as BYL719). We assume that high activation of HER2/HER3 can overcome the inhibitory effect of Alpelisib, and reactivate PI3K.

f_PIP3_=(PI3K or PI3K_2) and not PTEN

f_PIP3_2_=PI3K_2 and not PTEN

PI3K catalyzes the phosphorylation of PIP2 into PIP3 (PMID: 20622047). PTEN catalyzes the reverse process (PMID: 20622047). We assume that in the absence of PTEN the level of PIP3 follows the level of PI3K.

f_PDK1_pm_=PIP3 or PIP3_2

PIP3 recruits PDK1 to the membrane (PMID: 9895304).

f_mTORC2_pm_=(PIP3 or PIP3_2) and not Everolimus

mTORC2 is stimulated by PIP3 and directed to be co-localized with AKT in the plasma membrane (PMID: 24385483). It is inhibited by Everolimus.

f_AKT_=(PIP3 or PIP3_2) and (PDK1_pm or mTORC2_pm) and (not Ipatasertib or PIP3_2)

PIP3 recruits AKT to the membrane (PMID: 9895304). Membrane-bound PDK1 and mTORC2 phosphorylate AKT at two different sites (PMID: 9094314, PMID: 15718470). We assume that either activation mechanism is sufficient to AKT following its localization directed by PIP3. The drug Ipatasertib inhibits AKT activity; we assume that this inhibition can be overcome by a high level of PIP3. This is consistent with the observation that AKT activity recovers 24 hours after drug treatment in HER2+ cells (PMID: 21215704, PMID 24436048).

f_p21_p27_T_=FOXO3 or not (MYC_2 or MYC)

FOXO3 transcriptionally upregulates p27 (PMID: 10783894). MYC transcriptionally downregulates p21 and p27 (PMID: 15757889, PMID: 11313917). We assume that there is a base level of transcription of p21 or p27 that happens in the absence of MYC activity, thus we combine the positive and negative regulator with the “or not” rule.

f_p21_p27_=(not AKT and not PIM) or p21_p27_T

AKT phosphorylates p21 and inhibits its activity (PMID 11463845). PIM phosphorylates and inhibits the activity of p21 and p27 (PMID: 12431783, PMID:18593906). We assume that a sufficient amount of active (unphosphorylated) p21 or p27 is present if either of two conditions is satisfied: (i) AKT and PIM are absent or (ii) p21 or p27 transcription is upregulated, thus the total and with it the unphosphorylated p21 and/or p27 level increases.

f_cycE_CDK2_T_=E2F or E2F_2 or E2F_3

f_cycE_CDK2_=not p21_p27 and cycE_CDK2_T

E2F transcriptional activators activate the transcription of S phase promoting genes (PMID: 11257102). p21 and/or p27 inhibit CDK2 (PMID: 10385618)

f_KMT2D_=not AKT

Phosphorylation by AKT attenuates KMT2D’s methyltransferase activity (PMID 28336670)

f_TSC_=not AKT and not SGK1 and not MAPK_2

Both AKT and SGK phosphorylates TSC2, which leads to its destabilization (PMID: 12172553, PMID: 27451907). High ERK activation also inactivates TSC2 (PMID: 17671177).

f_PRAS40_=not AKT and not PIM

Both AKT and PIM phosphorylate PRAS40 , which prevents its binding to mTORC1 (PMID: 17386266, PMID: 27604488).

f_mTORC1_=(not TSC or not PRAS40) and not Everolimus

PRAS40 is a component and negative regulator of mTORC1, PMID: 17386266. TSC2 inhibits mTORC1 activation (through converting Rheb to its inactive, GTP-bound form, PMID: 12172553). We assume that mTORC1 is active if not targeted by Everolimus and if either inhibitor is inactive.

f_FOXO3_=(not AKT and not SGK1 and not PIM) and not FOXO3_Ub

f_FOXO3_Ub_=MAPK_2

AKT, SGK1 and PIM phosphorylate FOXO3, which leads to its inhibition (PMID: 10102273, PMID: 27451907, PMID:18593906). High ERK activity leads to the ubiquitin-tagging and then proteosomal degradation of FOXO3 (PMID: 18204439). We represent the status of the FOXO3 ubiquitin proteasomal degradation as the FOXO3_Ub node. This node is among the slow nodes (i.e. it is updated with a lower probability).

f_BIM_=(FOXO3 and not MAPK_2) or BIM_T

FOXO3 activates BIM, PMID: 11050388. ERK phosphorylates BIM, which leads to its proteasomal degradation, PMID: 14555991; we assume that this effect happens at high ERK activity only.

f_BAD_= not AKT and not PIM1 and not (MAPK or MAPK_2)

AKT, PIM1 and ERK phosphorylate BAD, which leads to its binding to 14-3-3 proteins and sequestration (PMID: 16226704, PMID: 27604488, PMID: 16226704).

f_MCL1_= translation

MCL-1 is translated in a cap-dependent manner (PMID: 27974663). We assume that this is the main mode of regulation of this anti-apoptotic protein.

f_EIF4F_=mTORC1

mTORC1 (through phosphorylating 4EBP1) allows EIF4E to initiate cap-dependent translation, PMID: 19339977

f_S6K_=mTORC1

mTORC1 activates S6K, PMID: 19339977

f_translation_=EIF4F and S6K

EIF4F initiates cap-dependent translation, PMID: 19339977. S6K activity leads to an increase in mRNA biogenesis and cap-dependent translation, PMID: 19339977.

f_ESR1_=(ER or FOXO3) and not Fulvestrant

f_ESR1_2_=ER and FOXO3 and not Fulvestrant

Cells can have an intrinsic ESR1 level, reflected in the variable ER. Alternatively, FOXO3A can bind to the ESR1 promoter and promote transcription, PMID: 25877889. Fulvestrant is a drug inhibitor of ESR1.

f_FOXA1_= FOXO3

We assume that the pioneer factor FOXA1 is upregulated by FOXO3. This is supported by the existence of a FOXO3 binding site in the promoter region of FOXA1 (based on DECODE by SABiosciences, QIAGEN; http://www.sabiosciences.com/chipqpcrsearch.php?species_id=0&nfactor=n&ninfo=n&ngene=n&B2=Search&src=genecard&factor=Over+200+TF&gene=FOXA1).

f_ER_transcription_=ER and (ESR1 or ESR1_2)

f_ER_transcription_2_=KMT2D and FOXA1 and PBX1 and ER and ESR1_2

The transcriptional regulatory activity of ER is highly enhanced by an open chromatin state (mediated by the histone H3 methyltransferase KMT2D) and the binding of the co-activators FOXA1 and PBX1 (PMID: 28336670)

f_MYC_=ER_transcription

f_MYC_2_=ER_transcription_2

MYC is a key ER-dependent transcriptional target, PMID: 11136970.

f_cyclinD_=MYC

f_cyclinD_2_=MYC_2

MYC activates the transcription of CDK4 and of cyclin D, PMID: 10688915

f_BCL2_=ER_transcription_2 or BCL2_T

In addition to intrinsic expression (incorporated in the source node BCL2_T), BCL2 can be upregulated by ER-dependent transcription (PMID: 21677677, PMID: 20154269, PMID: 11738551).

f_CDK4/6_=not Palbociclib

f_cycD_CDK4/6_=(cyclinD or cyclinD_2) and CDK4/6

f_cycD_CDK4/6_2_=(cyclinD_2) and CDK4/6

CDK4/6 is present unless inhibited by Palbociclib. CDK4/6 forms a complex with the D-type cyclins (PMID: 7610482); we assume that the complex has a higher activity when cyclinD has a higher activity.

f_pRb_=(cycD_CDK4/6_2 or cycD_CDK4/6) or cycE_CDK2

f_pRb_2_=(cycD_CDK4/6 and cycE_CDK2) or cycD_CDK4/6_2

f_pRb_3_=cycD_CDK4/6_2 and cycE_CDK2

CDK4/6 hyper-phosphorylates and inhibits Rb (PMID: 11257102). The cyclinE/CDK 2 kinase co-operates with cyclin D/CDK4/6 to phosphorylate Rb (PMID: 10323868). The highest level of hyper-phosphorylation is when both are at their most active levels.

f_E2F_=pRb

f_E2F_2_=pRb_2

f_E2F_3_=pRb_3 or (pRb_2 and E2F_3)

Unphosphorylated Rb binds to the E2F family of transcription factors, preventing it from interacting with the cell's transcription machinery (PMID: 11257102). Thus the (hyper)phosphorylated forms of Rb activate E2F. High E2F activity can be sustained by self-regulation, as E2F family members can activate their own transcription (PMID: 21677677, PMID: 18364697).

f_proliferation_=translation or E2F or E2F_2 or E2F_3

f_proliferation_2_=translation or E2F_2 or E2F_3

f_proliferation_3_=(translation and E2F_2) or E2F_3

f_proliferation_4_=translation and E2F_3

The output node proliferation focuses on two aspects of cell proliferation: cell cycle transition from G1 to S (reflected in the level of E2F), and protein translation. The lowest level of proliferation propensity is assumed in the presence of either of these; the highest corresponds to the simultaneous presence of protein translation and the highest activity level of E2F.

f_apoptosis_=(BIM and not (MCL1 and BCL2)) or (BIM and BAD) or (BAD and not (MCL1 and BCL2)) or apoptosis

f_apoptosis_2_=(BIM and BAD and not (MCL1 and BCL2)) or apoptosis_2

f_apoptosis_3_=((BIM and BAD and not (MCL1 or BCL2))) or apoptosis_3

The anti-apoptotic proteins BCL2 and MCL1 inhibit apoptosis effectors, while the apoptosis sensitizer BAD and the apoptosis activator BIM activate apoptosis effectors (PMID: 25723171, PMID: 25895919). We assume the lowest level of apoptosis propensity when the activation of a pro-apoptotic protein is coupled with the inactivity of one of the anti-apoptotic proteins, or alternatively when both types of pro-apoptotic proteins are active. The highest level of apoptosis propensity corresponds to the case when both types of pro-apoptotic proteins are active and both anti-apoptotic proteins are inactive. We assume that the apoptosis propensity cannot decrease (i.e. the apoptosis priming process is irreversible).

Initial or externally controlled states

The source nodes that correspond to drug treatment (Alpelisib, Fulvestrant, Neratinib, Palbociclib, Everolimus, Trametinib, Ipatasertib) are switched on according to the modeled treatment scenario.

Within the 13 source nodes, IGF1R_T, CDK4/6, and PBX1 are assumed to be ON, assuming an above-threshold intrinsic level. ER is assumed to be ON, HER2 and HER3_T are OFF, to reflect an ER+ /HER2- cell. PTEN, SGK1_T, PIM, PDK1, and mTORC2 (which act as resistance mechanisms to PI3K inhibitors) are OFF in the default case and assumed to be ON when simulating the relevant resistance scenario. The apoptotic protein transcripts BIM_T and BCL2_T can be either ON or OFF.

The long-term behaviors (attractors) of the model under the selected source node states are obtained using stable motif analysis (PMID: 25849586), which results in 8 steady state attractors: 6 cancerous steady states with high survivability ($Apoptosis =0$, $Proliferation=3$ or $4$) and 2 cancerous steady states with lower survivability ($Apoptosis =1$, $Proliferation=3$ or $4$). The initial state during simulations corresponds to a mix of two of the high survivability cancerous states (see Fig. 4A): PI3K and PIP3, AKT, RAS and MAPK is 1. FOXO3, PRAS40 and TSC are OFF. mTORC1 and the rest of the mTORC1 pathway are ON. ER_transcription, MYC and cyclinD, CDK4/6, and cycD_CDK4/6 are at level 1. p21/p27 and p21/p27_T are OFF, cycE/CDK2 and cycE/CDK2_T are OFF, Rb is phosphorylated, E2F is at level 2, and proliferation is at level 3. BAD is OFF, MCL1 is ON, BIM and BCL2 are simultaneously ON in 1/3 of the simulations (and OFF in the rest), apoptosis is at level 0.

Update probability of the nodes

The source nodes that correspond to drug treatment (Alpelisib, Fulvestrant, Neratinib, Palbociclib, Everolimus, Trametinib, Ipatasertib) are switched on according to the modeled treatment scenario.

We categorize nodes into fast or slow depending on whether the node is activated by a (fast) signaling event or a (slow) transcriptional/translational event, and set the update probability of fast nodes to be 5 times higher than that of slow nodes. To aid model expansion, this categorization includes source nodes whose regulatory function is $f_{X}= \sigma_{X}$ in the current version of the model.

The fast nodes are: AKT, BAD, cycE_CDK2, cycD_CDK4/6, EIF4F, FOXO3, HER2/HER3, KMT2D, MAPK, mTORC1, mTORC2_pm, p21_p27, PDK1_pm, PI3K, PIM, PIP3, PRAS40, RAS, S6K, SGK1, Translation, TSC.

The slow nodes are: Apoptosis, BCL2, BCL2_T, BIM, BIM_T, CDK4/6, cycE_CDK2_T, cyclinD, E2F, ER, ER_transcription, ESR1, FOXA1, FOXO3_Ub, HER2, HER3, HER3_T, IGF1R, IGF1R_T, MCL1, mTORC2, MYC, p21_p27_T, PBX1, PDK1, pRb, Proliferation, PTEN, SGK1_T.
