# Supplementary material for: A network modeling approach to elucidate drug resistance mechanisms and predict combinatorial drug treatments in breast cancer
Source: Cancer Converg. 2017 Dec 29;1(1):5. doi: 10.1186/s41236-017-0007-6 (PMC5876695; doi:10.1186/s41236-017-0007-6)
Supplement: Supplementary file 2 — Timing-dependent combinatorial effect of PI3K inhibitors and mTOR inhibitors. (DOCX 11 kb) [file 41236_2017_7_MOESM2_ESM.docx]

Table S1. Sequential synergy of PI3K inhibitors (Alpelisib) and mTOR inhibitors (Everolimus). We demonstrate that the combinatorial effect on apoptosis depends on the relative timing of the mTOR and PI3K inhibition by doing simulations in which we add the mTOR inhibitor at varying times before the PI3K inhibitor. The inhibitor applied first starts at t=0 and the second is delayed by 5, 10, 15 or 20 time steps; both inhibitors continue until the end of the simulations, 100 time steps after the second inhibitor is applied, which was found to be enough for the simulations to reach steady state.

| Time Alpelisib - time Everolimus | $\mathrm{Apoptosis}_{\mathrm{norm}}$ |
| --- | --- |
| MCL1=OFF | 0.75 |
| 20.00 | 0.75 |
| 15.00 | 0.75 |
| 10.00 | 0.75 |
| 5.00 | 0.74 |
| 0.00 | 0.73 |
| -5.00 | 0.71 |
| -10.00 | 0.70 |
| Alpelisib alone | 0.70 |
